# (η^5^-Cyclo­penta­dien­yl){[3-(2,2-dicyano­ethen­yl)bicyclo­[2.2.1]hepta-2,5-dien-2-yl]ethyn­yl}(triphenyl­phosphine)nickel(II)

**DOI:** 10.1107/S1600536808002328

**Published:** 2008-01-25

**Authors:** John F. Gallagher, Peter Butler, A. R. Manning

**Affiliations:** a School of Chemical Sciences, Dublin City University, Dublin 9, Ireland; b Department of Chemistry, University College Dublin, Belfield, Dublin 4, Ireland

## Abstract

The title compound, [Ni(C_5_H_5_)(C_13_H_7_N_2_)(C_18_H_15_P)] or (η^5^-C_5_H_5_)(PPh_3_)Ni—C C—C_7_H_6_—C(H)=C(CN)_2_, contains an unusual disubstituted norbornadienyl (NBD) ligand containing ethynyl (–C C–) and dicyano­vinyl [–C(H)=C(CN)_2_] groups. Disorder is present in the NBD group with site occupancies of 0.636 (10) and 0.364 (10) for two distinct orientations. There are no strong hydrogen bonds and the primary inter­actions are weak C—H⋯π(arene) inter­actions.

## Related literature

For related literature, see: Butler *et al.* (1998 ▶, 2005 ▶, 2007 ▶); Gallagher *et al.* (1998 ▶, 2002 ▶); McArdle (1995 ▶); Whittal *et al.* (1998*a* ▶,*b* ▶).
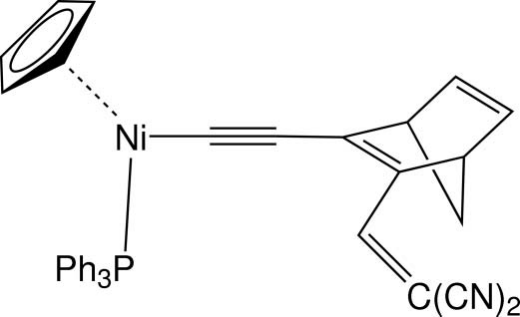


## Experimental

### 

#### Crystal data

[Ni(C_5_H_5_)(C_13_H_7_N_2_)(C_18_H_15_P)]
*M* *_r_* = 577.28
Triclinic, 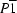

*a* = 10.7972 (16) Å
*b* = 11.8155 (14) Å
*c* = 12.1248 (14) Å
α = 73.169 (5)°
β = 78.153 (9)°
γ = 78.586 (9)°
*V* = 1433.1 (3) Å^3^
*Z* = 2
Mo *K*α radiation
μ = 0.76 mm^−1^
*T* = 296 (1) K
0.50 × 0.40 × 0.30 mm

#### Data collection

Bruker P4 diffractometer
Absorption correction: ψ scan (North *et al.*, 1968 ▶) *T*_min_ = 0.716, *T*_max_ = 0.883 (expected range = 0.645–0.796)
7832 measured reflections
6787 independent reflections
4533 reflections with *I* > 2σ(*I*)
*R*_int_ = 0.029
3 standard reflections every 197 reflections intensity decay: 5%

#### Refinement

*R*[*F* ^2^ > 2σ(*F* ^2^)] = 0.047
*wR*(*F* ^2^) = 0.108
*S* = 1.01
6787 reflections
407 parameters
94 restraints
H-atom parameters constrained
Δρ_max_ = 0.33 e Å^−3^
Δρ_min_ = −0.25 e Å^−3^

### 

Data collection: *XSCANS* (Bruker, 1996 ▶); cell refinement: *XSCANS*; data reduction: *XSCANS*; program(s) used to solve structure: *SHELXS97* (Sheldrick, 2008 ▶); program(s) used to refine structure: *SHELXL97* (Sheldrick, 2008 ▶); molecular graphics: *PLATON* (Spek, 2003 ▶); software used to prepare material for publication: *SHELXL97* and *PREP8* (Ferguson, 1998 ▶).

## Supplementary Material

Crystal structure: contains datablocks global, I. DOI: 10.1107/S1600536808002328/lh2592sup1.cif

Structure factors: contains datablocks I. DOI: 10.1107/S1600536808002328/lh2592Isup2.hkl

Additional supplementary materials:  crystallographic information; 3D view; checkCIF report

## Figures and Tables

**Table 1:** Hydrogen-bond geometry (Å, °)

*D*—H⋯*A*	*D*—H	H⋯*A*	*D*⋯*A*	*D*—H⋯*A*
C33—H33⋯*Cg*1^i^	0.93	2.98	3.648 (4)	130

Symmetry code: (i) 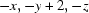. *Cg*1 is the centroid of the cyclo­penta­dienyl ring.

**Fig. 1.**
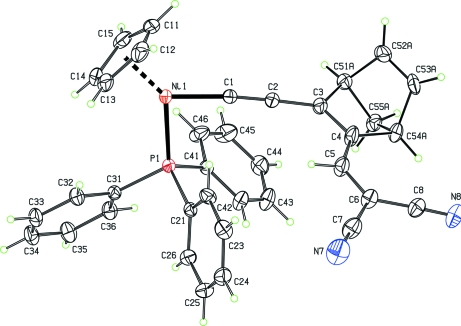
A view of the major conformation of (I) with the atomic numbering scheme. Displacement ellipsoids are drawn at the 20% probability level.

**Fig. 2.**
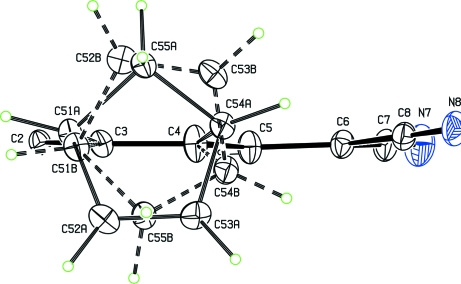
A view of the NBD disorder in (I): the (cp)(PPh3)Ni—C atoms are omitted for clarity. Displacement ellipsoids are drawn at the 20% probability level.

**Fig. 3.**
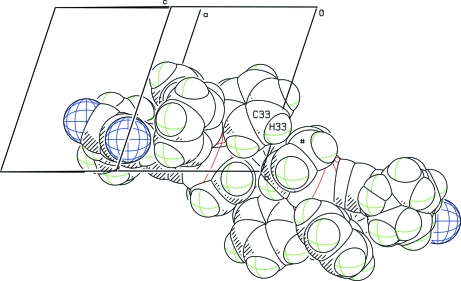
A view of the principal C—H···π(arene) interaction and unit cell in (I) with atoms depicted as their van der Waals spheres. The atom labels C33—H33 and # (cp ring) signify the interaction listed in Table 1.

## supplementary crystallographic information

### Comment

The acetylide linkage in Ni(η^5^-C_5_H_5_)(PPh_3_)-C≡C—*X* complexes allows facile electronic communication between the electron rich Ni(η^5^-C_5_H_5_)(PPh_3_) moiety and the *X* group (*X* = alkyl, arene) thus affecting the characteristic chemistry of both *X* and the acetylide linkage (Gallagher *et al.*, 2002). However, if *X* is an electron withdrawing group the molecule is a donor-π-acceptor (D-π-A) system which may have non-linear optical (NLO) properties (Whittal *et al.*, 1998*a*,b) although the phenyl derivative (*X* = C_6_H_5_) does not appear to be particularly effective.

We have demonstrated that polycylic hydrocarbons containing 1–5 aromatic rings can act as an electron-donor endgroup in D-π-A systems in the presence of suitable acceptors and have examined their behaviour when attached to the Ni(η^5^-C_5_H_5_)(PPh_3_) donor moiety (Butler *et al.*, 2005, 2007). The spectroscopic and electrochemical evidence suggests limited communication between either end of these Ni(η^5^-C_5_H_5_)(PPh_3_)-C≡C—*X* systems at least in the ground state and is not sufficient to influence significant changes in the geometric data from diffraction measurements. Herein, we present an unusual norbornadiene derivative (I) (η^5^-C_5_H_5_)(PPh_3_)Ni—C≡C-NBD-C(H)=C(CN)_2_ (where NBD is a 2,3-substituted C_7_H_6_ group).

Molecule (I) has a half sandwich structure at the Ni^II^ centre and contains the σ-bonded ethynyl-2-norbornadienyl(methylidene)propanedinitrile ligand, a η^5^-C_5_H_5_ ring and triphenylphosphine bonded to the Ni^II^ atom. A view of the molecule with atomic numbering scheme is depicted (Fig. 1). The principal Ni-ligand dimensions include Ni1—P1 2.1417 (8) Å, Ni1—C1 1.839 (3) Å, Ni···*Cg* 1.7444 (16) Å (*Cg* is the cyclopentadienyl ring centroid), P1—Ni1—C1 87.86 (8)° and similar to geometric data in related derivatives (Gallagher *et al.*, 1998, 2002; Butler *et al.*, 1998, 2005). The acetylide –C≡C– and *_sp_*C—C_NBD_ bond lengths are 1.214 (4) Å and 1.403 (4) Å and similar to the geometric data reported for the dicyanovinyl derivative (II) (η^5^-C_5_H_5_)(PPh_3_)Ni—C≡C—C(H)=C(C≡N)_2_ (Gallagher *et al.*, 2002). The two C=C bond lengths of 1.371 (4) Å (C3=C4) and 1.358 (4) Å (C5=C6) can be explained by an increase of delocalization along the conjugated metallo-ligand chain and the increase in bond lengths often observed in strained ring systems *i.e.* the C3=C4 in the NBD ring system.

The Ni—C≡C—C chain bond angles deviate slightly from linearity with Ni—C≡C 173.6 (2)° and C≡C—C 171.8 (3)°: this is greater than the two corresponding 176.6 (2)°/177.8 (3)° angles reported for (II) but similar to related systems (Butler *et al.*, 1998) and this can be attributed mainly to crystal packing forces.

The η^5^-C_5_H_5_ ring is orthogonal to the P1/Ni1/C1 plane, 88.84 (10)°. Of interest is the relative co-planarity of atoms in the 11-atom chain Ni1—C1≡C2—C3=C4—C5=C6(C≡N)_2_, where the Ni1 and C8 atoms deviate by a maximum of 0.334 (2) Å and 0.269 (2) Å from the 11-atom plane (the next greatest deviation is C2 by 0.159 (3) Å). This highlights the relative co-planarity of atoms along this chain increasing the potential for conjugation effects.

The closest intramolecular contact to Ni1 involves H22 with H22···Ni1 2.94 Å and C22—H22···Ni1 119° (C22 is the closest PPh_3_*ortho*-C to Ni1 at 3.476 (3) Å). The three Ni1—P—C angles vary as 111.28 (9)°, 113.62 (9)° and 118.31 (8)°. There is a small asymmetry in the PPh_3_ ligand with four P—C—C angles in the range 119.1 (2)° to 122.5 (2)° at C21 and C31. However, at C41 these P—C—C angles are 117.53 (2)° and 124.5 (2)°. The three P—C*_ipso_*···C*_para_* angles are 179.50 (16)°, 177.56 (15)°, 175.90 (16)° and reflecting the greater phenyl asymmetry at C41.

In the absence of strong hydrogen bond donors or acceptors, C—H···π(arene) interactions involving the PPh_3_ arene rings arise (details in Table) (Fig. 2). The C33···{C11,···,C15} distances are in the range 3.618 (4) Å to 4.023 (4) Å and C—H···C angles (in *Cg*1) vary from 108° to 147°.

### Experimental

Compound (I) was prepared according to literature methods (Butler *et al.*, 1998, 2005, 2007), (Gallagher *et al.*, 2002) and involves hydrolysis of an acetal precursor to an aldehyde and subsequent reaction with malonitrile H_2_C(C≡N)_2_ to give the dicyanovinyl derivative (title compound). Yield 95%. Crystals suitable for X-ray diffraction were grown from Et_2_O/hexane. ^1^H NMR (δ, 270 MHz, CDCl_3_): 7.68 - 7.37 (m, 15H, PPh_3_), 6.68 (s, 1H, –CH=), 6.63 and 6.29 (d, 2H, –C(H)=C(H)-), 5.26 (s, 5H, Cp), 4.31, 3.04 (s, 2H, bridgehead H), 1.78 (dd, ^1^J_HH_ = 8 Hz, 2H, µ-CH_2_). ^13^C NMR (δ, 270 MHz, CDCl_3_): 165.81 (s, Ni—C≡C), 148.59 and 147.43 (s, C_3_ and C_4_), 142.61 and 139.89 (s, C_52 A/B_ and C_53 A/B_), 132.95 (d, ^1^J_CP_ = 50 Hz, Ni—C), 133.7 - 128.4 (m, PPh_3_), 119.33 (s, C(C≡N)_2_, 116.59 and 115.37 (s, C≡N), 93.27 (s, Cp), 67.7 (s, µ-CH_2_), 57.85 and 47.22 (s, bridgehead C). IR (ν_C__≡__C_, cm^-1^): 2150 (CH_2_Cl_2_); 2152 (KBr) and (ν_C__≡__N_, cm^-1^): 2216 (CH_2_Cl_2_); 2216 (KBr). Microanalysis: calculated for C_36_H_21_N_2_PNi: C, 74.9, H, 4.7, N 4.8; found: C, 74.6, H, 5.2, N 4.8.

### Refinement

In the penultimate stages of refinement it was observed that there was disorder within the norbornadienyl (NBD) –C_7_H_6_– moiety: this was resolved and successfully modelled into two partial occupancy A/B residues [C51A/B,···,C55A/B] involving the –C_5_H_6_– bridgehead group as two A/B components with site occupancies of 0.636 (10):0.364 (10). The C3=C4 atoms were used as 'anchor' atoms for loose *DFIX* restraints: DELU/ISOR restraints were also used for the anisotropic displacement parameters (McArdle, 1995; Sheldrick, 2008). The orientational disorder is explained by swapping the NBD group –CH_2_ and –CH=CH– atoms.

All H atoms attached to aromatic C atoms were treated as riding atoms using the *SHELXL97* (Sheldrick, 2008) defaults at 296 (1) K, with C—H distances of 0.93 Å (for aromatic H) and in the range 0.93 to 0.98 Å (for aliphatic C—H) and with *U*_iso_(H) = 1.2*U*_eq_(C) for all H atoms.

### Crystal data

**Table:**

[Ni(C_5_H_5_)(C_13_H_7_N_2_)(C_18_H_15_P)]	*Z* = 2
*M**_r_* = 577.28	*F*_000_ = 600
Triclinic, *P*1	*D*_x_ = 1.338 Mg m^−^^3^
Hall symbol: -P 1	Mo *K*α radiation λ = 0.71073 Å
*a* = 10.7972 (16) Å	Cell parameters from 40 reflections
*b* = 11.8155 (14) Å	θ = 3.6–14.9º
*c* = 12.1248 (14) Å	µ = 0.76 mm^−^^1^
α = 73.169 (5)º	*T* = 296 (1) K
β = 78.153 (9)º	Block, green
γ = 78.586 (9)º	0.50 × 0.40 × 0.30 mm
*V* = 1433.1 (3) Å^3^	

### Data collection

**Table:**

Bruker P4 diffractometer	*R*_int_ = 0.029
Radiation source: X-ray tube	θ_max_ = 28.0º
Monochromator: graphite	θ_min_ = 2.0º
*T* = 296(1) K	*h* = −1→14
ω scans	*k* = −14→15
Absorption correction: ψ scan(North *et al.*, 1968)	*l* = −15→16
*T*_min_ = 0.716, *T*_max_ = 0.883	3 standard reflections
7832 measured reflections	every 197 reflections
6787 independent reflections	intensity decay: 5%
4533 reflections with *I* > 2σ(*I*)	

### Refinement

**Table:**

Refinement on *F*^2^	Secondary atom site location: difference Fourier map
Least-squares matrix: full	Hydrogen site location: inferred from neighbouring sites
*R*[*F*^2^ > 2σ(*F*^2^)] = 0.047	H-atom parameters constrained
*wR*(*F*^2^) = 0.108	*w* = 1/[σ^2^(*F*_o_^2^) + (0.0422*P*)^2^ + 0.2761*P*] where *P* = (*F*_o_^2^ + 2*F*_c_^2^)/3
*S* = 1.01	(Δ/σ)_max_ = 0.001
6787 reflections	Δρ_max_ = 0.33 e Å^−^^3^
407 parameters	Δρ_min_ = −0.25 e Å^−^^3^
94 restraints	Extinction correction: none
Primary atom site location: structure-invariant direct methods	

### Fractional atomic coordinates and isotropic or equivalent isotropic displacement parameters (Å^2^)

**Table:**

	*x*	*y*	*z*	*U*_iso_*/*U*_eq_	Occ. (<1)
Ni1	0.27893 (3)	0.93823 (3)	0.16592 (3)	0.04182 (11)	
P1	0.25181 (6)	0.75680 (6)	0.25072 (6)	0.03890 (16)	
C7	0.3069 (4)	0.8014 (3)	0.7760 (3)	0.0742 (10)	
N7	0.2068 (4)	0.8081 (4)	0.8281 (3)	0.1087 (13)	
C8	0.5323 (3)	0.7390 (3)	0.7788 (3)	0.0656 (9)	
N8	0.6099 (3)	0.6948 (3)	0.8358 (3)	0.0900 (10)	
C11	0.3133 (4)	1.0983 (3)	0.0448 (3)	0.0775 (11)	
C12	0.1960 (4)	1.1199 (3)	0.1177 (3)	0.0754 (10)	
C13	0.1155 (3)	1.0515 (3)	0.1036 (3)	0.0673 (9)	
C14	0.1824 (3)	0.9861 (3)	0.0235 (3)	0.0630 (9)	
C15	0.3020 (3)	1.0210 (3)	−0.0173 (3)	0.0713 (10)	
C21	0.1743 (2)	0.7343 (2)	0.4027 (2)	0.0411 (6)	
C22	0.1526 (3)	0.8261 (3)	0.4567 (2)	0.0525 (7)	
C23	0.0924 (3)	0.8097 (3)	0.5709 (3)	0.0641 (8)	
C24	0.0546 (3)	0.7015 (3)	0.6334 (3)	0.0654 (9)	
C25	0.0729 (3)	0.6103 (3)	0.5809 (3)	0.0628 (8)	
C26	0.1314 (3)	0.6267 (3)	0.4661 (2)	0.0538 (7)	
C31	0.1525 (2)	0.6869 (2)	0.1912 (2)	0.0404 (6)	
C32	0.0282 (3)	0.7414 (3)	0.1783 (2)	0.0510 (7)	
C33	−0.0502 (3)	0.6905 (3)	0.1360 (3)	0.0564 (8)	
C34	−0.0066 (3)	0.5840 (3)	0.1077 (3)	0.0621 (8)	
C35	0.1157 (3)	0.5280 (3)	0.1206 (3)	0.0638 (8)	
C36	0.1958 (3)	0.5797 (3)	0.1611 (2)	0.0537 (7)	
C41	0.4038 (2)	0.6565 (2)	0.2487 (2)	0.0416 (6)	
C42	0.4416 (3)	0.5703 (3)	0.3439 (3)	0.0588 (8)	
C43	0.5589 (3)	0.4972 (3)	0.3331 (3)	0.0739 (10)	
C44	0.6380 (3)	0.5098 (3)	0.2283 (3)	0.0677 (9)	
C45	0.6005 (3)	0.5934 (3)	0.1337 (3)	0.0832 (12)	
C46	0.4849 (3)	0.6665 (3)	0.1434 (3)	0.0703 (10)	
C1	0.3981 (2)	0.9205 (2)	0.2607 (2)	0.0437 (6)	
C2	0.4765 (3)	0.8968 (2)	0.3259 (2)	0.0474 (6)	
C3	0.5745 (2)	0.8557 (3)	0.3944 (2)	0.0521 (7)	
C4	0.5668 (3)	0.8212 (3)	0.5134 (3)	0.0717 (10)	
C5	0.4523 (3)	0.8275 (3)	0.5920 (3)	0.0650 (9)	
C6	0.4332 (3)	0.7919 (3)	0.7104 (3)	0.0607 (8)	
C51A	0.7154 (7)	0.8267 (12)	0.3422 (11)	0.055 (3)	0.636 (10)
C52A	0.7821 (10)	0.9071 (9)	0.3796 (7)	0.067 (2)	0.636 (10)
C53A	0.7758 (7)	0.8686 (6)	0.4953 (7)	0.067 (2)	0.636 (10)
C55A	0.7544 (17)	0.7069 (12)	0.4307 (8)	0.055 (2)	0.636 (10)
C54A	0.7055 (5)	0.7614 (7)	0.5348 (6)	0.0548 (19)	0.636 (10)
C51B	0.7190 (9)	0.841 (2)	0.3499 (18)	0.055 (6)	0.364 (10)
C52B	0.754 (3)	0.709 (2)	0.4038 (14)	0.070 (6)	0.364 (10)
C53B	0.7446 (10)	0.6940 (10)	0.5189 (12)	0.065 (3)	0.364 (10)
C55B	0.7770 (16)	0.8884 (15)	0.4301 (11)	0.056 (4)	0.364 (10)
C54B	0.7028 (9)	0.8154 (11)	0.5398 (10)	0.064 (4)	0.364 (10)
H11	0.3866	1.1310	0.0395	0.093*	
H12	0.1772	1.1712	0.1663	0.090*	
H13	0.0312	1.0485	0.1404	0.081*	
H14	0.1513	0.9291	0.0017	0.076*	
H15	0.3630	0.9968	−0.0755	0.086*	
H22	0.1787	0.8994	0.4158	0.063*	
H23	0.0772	0.8724	0.6060	0.077*	
H24	0.0166	0.6905	0.7112	0.078*	
H25	0.0462	0.5375	0.6224	0.075*	
H26	0.1424	0.5648	0.4304	0.065*	
H32	−0.0026	0.8131	0.1986	0.061*	
H33	−0.1328	0.7286	0.1267	0.068*	
H34	−0.0599	0.5495	0.0798	0.074*	
H35	0.1448	0.4552	0.1021	0.077*	
H36	0.2791	0.5423	0.1681	0.064*	
H42	0.3886	0.5607	0.4159	0.071*	
H43	0.5838	0.4390	0.3980	0.089*	
H44	0.7170	0.4615	0.2220	0.081*	
H45	0.6533	0.6015	0.0617	0.100*	
H46	0.4607	0.7238	0.0776	0.084*	
H5	0.3790	0.8608	0.5582	0.078*	
H51A	0.7338	0.8274	0.2595	0.066*	0.636 (10)
H52A	0.8206	0.9716	0.3310	0.081*	0.636 (10)
H53A	0.8084	0.9013	0.5425	0.080*	0.636 (10)
H55A	0.7084	0.6438	0.4318	0.066*	0.636 (10)
H55B	0.8459	0.6800	0.4210	0.066*	0.636 (10)
H54A	0.7147	0.7083	0.6123	0.066*	0.636 (10)
H51B	0.7462	0.8700	0.2657	0.066*	0.364 (10)
H52B	0.7767	0.6490	0.3646	0.085*	0.364 (10)
H53B	0.7612	0.6224	0.5747	0.078*	0.364 (10)
H55C	0.8690	0.8656	0.4247	0.067*	0.364 (10)
H55D	0.7539	0.9740	0.4199	0.067*	0.364 (10)
H54B	0.7105	0.8256	0.6154	0.077*	0.364 (10)

### Atomic displacement parameters (Å^2^)

**Table:**

	*U*^11^	*U*^22^	*U*^33^	*U*^12^	*U*^13^	*U*^23^
Ni1	0.03662 (18)	0.0448 (2)	0.04238 (19)	−0.00811 (14)	−0.01386 (14)	−0.00215 (14)
P1	0.0332 (3)	0.0436 (4)	0.0394 (3)	−0.0066 (3)	−0.0086 (3)	−0.0073 (3)
C7	0.077 (3)	0.086 (3)	0.0514 (19)	0.006 (2)	−0.0049 (18)	−0.0218 (18)
N7	0.084 (3)	0.150 (4)	0.079 (2)	0.007 (2)	0.006 (2)	−0.039 (2)
C8	0.079 (2)	0.068 (2)	0.0507 (18)	−0.0046 (18)	−0.0169 (17)	−0.0155 (16)
N8	0.105 (3)	0.099 (2)	0.0661 (19)	0.004 (2)	−0.0413 (19)	−0.0155 (17)
C11	0.071 (2)	0.061 (2)	0.088 (3)	−0.0264 (18)	−0.039 (2)	0.0289 (19)
C12	0.096 (3)	0.0448 (18)	0.084 (3)	0.0104 (18)	−0.044 (2)	−0.0085 (17)
C13	0.0409 (16)	0.077 (2)	0.069 (2)	0.0022 (16)	−0.0194 (15)	0.0032 (18)
C14	0.067 (2)	0.065 (2)	0.0586 (19)	−0.0101 (17)	−0.0347 (17)	−0.0017 (16)
C15	0.067 (2)	0.084 (2)	0.0422 (17)	−0.0023 (19)	−0.0077 (16)	0.0097 (16)
C21	0.0331 (13)	0.0500 (15)	0.0407 (14)	−0.0073 (11)	−0.0073 (11)	−0.0105 (12)
C22	0.0447 (15)	0.0619 (18)	0.0533 (17)	−0.0163 (13)	0.0014 (13)	−0.0192 (14)
C23	0.0578 (19)	0.084 (2)	0.0586 (19)	−0.0153 (17)	0.0001 (15)	−0.0341 (18)
C24	0.0547 (19)	0.092 (3)	0.0448 (17)	−0.0148 (17)	−0.0003 (14)	−0.0126 (17)
C25	0.065 (2)	0.065 (2)	0.0507 (18)	−0.0206 (16)	−0.0039 (15)	0.0004 (15)
C26	0.0585 (18)	0.0542 (17)	0.0488 (16)	−0.0136 (14)	−0.0090 (14)	−0.0095 (13)
C31	0.0359 (13)	0.0494 (15)	0.0358 (13)	−0.0089 (11)	−0.0054 (10)	−0.0092 (11)
C32	0.0391 (14)	0.0566 (17)	0.0605 (18)	−0.0020 (12)	−0.0106 (13)	−0.0216 (14)
C33	0.0375 (15)	0.077 (2)	0.0587 (18)	−0.0057 (14)	−0.0137 (13)	−0.0209 (16)
C34	0.0533 (18)	0.091 (2)	0.0549 (18)	−0.0209 (17)	−0.0097 (14)	−0.0317 (17)
C35	0.062 (2)	0.072 (2)	0.072 (2)	−0.0083 (16)	−0.0144 (16)	−0.0401 (18)
C36	0.0434 (15)	0.0634 (19)	0.0574 (18)	−0.0028 (13)	−0.0097 (13)	−0.0228 (15)
C41	0.0357 (13)	0.0417 (14)	0.0466 (15)	−0.0057 (11)	−0.0092 (11)	−0.0084 (11)
C42	0.0584 (18)	0.070 (2)	0.0435 (16)	0.0057 (15)	−0.0153 (14)	−0.0132 (14)
C43	0.070 (2)	0.078 (2)	0.068 (2)	0.0182 (18)	−0.0344 (19)	−0.0136 (18)
C44	0.0469 (17)	0.062 (2)	0.091 (3)	0.0065 (15)	−0.0169 (18)	−0.0219 (19)
C45	0.0493 (19)	0.088 (3)	0.078 (2)	0.0088 (18)	0.0132 (17)	0.002 (2)
C46	0.0476 (17)	0.073 (2)	0.061 (2)	0.0035 (16)	0.0039 (15)	0.0104 (16)
C1	0.0410 (14)	0.0420 (14)	0.0462 (15)	−0.0084 (11)	−0.0094 (12)	−0.0051 (12)
C2	0.0438 (15)	0.0529 (16)	0.0435 (15)	−0.0095 (12)	−0.0107 (12)	−0.0050 (12)
C3	0.0402 (15)	0.0603 (18)	0.0513 (16)	−0.0047 (13)	−0.0125 (13)	−0.0055 (14)
C4	0.0404 (16)	0.111 (3)	0.0523 (18)	0.0029 (17)	−0.0173 (14)	−0.0063 (18)
C5	0.0476 (17)	0.088 (2)	0.0522 (18)	0.0031 (16)	−0.0150 (14)	−0.0107 (17)
C6	0.0596 (19)	0.067 (2)	0.0518 (18)	0.0048 (15)	−0.0122 (15)	−0.0176 (15)
C51A	0.045 (6)	0.068 (6)	0.049 (5)	−0.012 (4)	−0.009 (4)	−0.007 (4)
C52A	0.041 (3)	0.067 (4)	0.086 (6)	−0.011 (3)	−0.016 (5)	−0.001 (5)
C53A	0.048 (4)	0.076 (4)	0.090 (6)	−0.008 (3)	−0.037 (4)	−0.025 (4)
C55A	0.046 (4)	0.051 (4)	0.060 (5)	0.008 (3)	−0.018 (4)	−0.007 (3)
C54A	0.044 (3)	0.054 (5)	0.060 (3)	0.018 (3)	−0.024 (3)	−0.012 (3)
C51B	0.035 (9)	0.061 (9)	0.060 (9)	0.009 (7)	−0.014 (7)	−0.010 (7)
C52B	0.059 (9)	0.070 (10)	0.081 (10)	−0.008 (7)	−0.010 (9)	−0.019 (8)
C53B	0.047 (6)	0.061 (6)	0.077 (7)	−0.005 (5)	−0.023 (5)	0.005 (6)
C55B	0.048 (6)	0.070 (8)	0.057 (9)	−0.019 (5)	−0.024 (8)	−0.007 (8)
C54B	0.068 (8)	0.061 (8)	0.056 (6)	0.014 (7)	−0.017 (5)	−0.016 (6)

### Geometric parameters (Å, °)

**Table:**

Ni1—C1	1.839 (3)	C23—H23	0.9300
Ni1—C11	2.078 (3)	C24—C25	1.366 (5)
Ni1—C12	2.116 (3)	C24—H24	0.9300
Ni1—C13	2.122 (3)	C25—C26	1.380 (4)
Ni1—C14	2.081 (3)	C25—H25	0.9300
Ni1—C15	2.136 (3)	C26—H26	0.9300
Ni1—P1	2.1417 (8)	C31—C36	1.385 (4)
P1—C21	1.827 (3)	C31—C32	1.388 (3)
P1—C31	1.835 (3)	C32—C33	1.378 (4)
P1—C41	1.824 (3)	C32—H32	0.9300
C1—C2	1.214 (3)	C33—C34	1.368 (4)
C2—C3	1.403 (4)	C33—H33	0.9300
C3—C4	1.371 (4)	C34—C35	1.373 (4)
C3—C51A	1.535 (7)	C34—H34	0.9300
C3—C51B	1.535 (9)	C35—C36	1.385 (4)
C4—C5	1.400 (4)	C35—H35	0.9300
C4—C54B	1.550 (9)	C36—H36	0.9300
C4—C54A	1.563 (6)	C41—C42	1.373 (4)
C5—C6	1.358 (4)	C41—C46	1.380 (4)
C5—H5	0.9300	C42—C43	1.390 (4)
C7—N7	1.135 (5)	C42—H42	0.9300
C7—C6	1.432 (5)	C43—C44	1.364 (5)
C8—N8	1.144 (4)	C43—H43	0.9300
C8—C6	1.423 (4)	C44—C45	1.354 (5)
C11—C15	1.377 (5)	C44—H44	0.9300
C11—C12	1.412 (5)	C45—C46	1.375 (4)
C11—H11	0.9300	C45—H45	0.9300
C12—C13	1.363 (5)	C46—H46	0.9300
C12—H12	0.9300	C51A—C52A	1.510 (8)
C13—C14	1.410 (5)	C51A—C55A	1.550 (7)
C13—H13	0.9300	C52A—C53A	1.335 (7)
C14—C15	1.382 (5)	C53A—C54A	1.514 (7)
C14—H14	0.9300	C55A—C54A	1.532 (8)
C15—H15	0.9300	C51B—C52B	1.510 (10)
C21—C22	1.381 (4)	C51B—C55B	1.536 (9)
C21—C26	1.394 (4)	C52B—C53B	1.340 (9)
C22—C23	1.379 (4)	C53B—C54B	1.493 (9)
C22—H22	0.9300	C55B—C54B	1.525 (9)
C23—C24	1.377 (5)		
			
C1—Ni1—C11	100.90 (12)	C25—C26—H26	119.4
C1—Ni1—C14	163.39 (12)	C21—C26—H26	119.4
C11—Ni1—C14	64.40 (14)	C36—C31—C32	118.4 (2)
C1—Ni1—C12	109.61 (13)	C36—C31—P1	122.5 (2)
C11—Ni1—C12	39.34 (14)	C32—C31—P1	119.1 (2)
C14—Ni1—C12	64.61 (14)	C33—C32—C31	120.8 (3)
C1—Ni1—C13	143.35 (14)	C33—C32—H32	119.6
C11—Ni1—C13	64.26 (13)	C31—C32—H32	119.6
C14—Ni1—C13	39.18 (13)	C34—C33—C32	120.1 (3)
C12—Ni1—C13	37.52 (13)	C34—C33—H33	119.9
C1—Ni1—C15	125.25 (13)	C32—C33—H33	119.9
C11—Ni1—C15	38.10 (14)	C33—C34—C35	120.0 (3)
C14—Ni1—C15	38.24 (13)	C33—C34—H34	120.0
C12—Ni1—C15	64.59 (15)	C35—C34—H34	120.0
C13—Ni1—C15	64.33 (13)	C34—C35—C36	120.1 (3)
C1—Ni1—P1	87.86 (8)	C34—C35—H35	119.9
C11—Ni1—P1	165.01 (13)	C36—C35—H35	119.9
C14—Ni1—P1	104.95 (10)	C35—C36—C31	120.4 (3)
C12—Ni1—P1	147.85 (12)	C35—C36—H36	119.8
C13—Ni1—P1	114.96 (10)	C31—C36—H36	119.8
C15—Ni1—P1	127.08 (12)	C42—C41—C46	117.9 (3)
C41—P1—C21	107.44 (12)	C42—C41—P1	124.5 (2)
C41—P1—C31	103.20 (12)	C46—C41—P1	117.5 (2)
C21—P1—C31	101.86 (11)	C41—C42—C43	120.2 (3)
C41—P1—Ni1	111.28 (9)	C41—C42—H42	119.9
C21—P1—Ni1	113.62 (9)	C43—C42—H42	119.9
C31—P1—Ni1	118.31 (8)	C44—C43—C42	120.8 (3)
N7—C7—C6	179.5 (4)	C44—C43—H43	119.6
N8—C8—C6	178.3 (4)	C42—C43—H43	119.6
C15—C11—C12	109.1 (3)	C45—C44—C43	119.4 (3)
C15—C11—Ni1	73.25 (19)	C45—C44—H44	120.3
C12—C11—Ni1	71.79 (19)	C43—C44—H44	120.3
C15—C11—H11	125.5	C44—C45—C46	120.4 (3)
C12—C11—H11	125.5	C44—C45—H45	119.8
Ni1—C11—H11	121.2	C46—C45—H45	119.8
C13—C12—C11	107.2 (3)	C45—C46—C41	121.4 (3)
C13—C12—Ni1	71.49 (19)	C45—C46—H46	119.3
C11—C12—Ni1	68.86 (18)	C41—C46—H46	119.3
C13—C12—H12	126.4	C2—C1—Ni1	173.6 (2)
C11—C12—H12	126.4	C1—C2—C3	171.8 (3)
Ni1—C12—H12	124.9	C4—C3—C2	129.7 (3)
C12—C13—C14	108.0 (3)	C4—C3—C51A	107.3 (5)
C12—C13—Ni1	70.99 (18)	C2—C3—C51A	122.6 (5)
C14—C13—Ni1	68.82 (16)	C4—C3—C51B	103.7 (8)
C12—C13—H13	126.0	C2—C3—C51B	126.6 (8)
C14—C13—H13	126.0	C3—C4—C5	124.3 (3)
Ni1—C13—H13	125.7	C3—C4—C54B	106.7 (5)
C15—C14—C13	108.5 (3)	C5—C4—C54B	126.2 (5)
C15—C14—Ni1	73.05 (18)	C3—C4—C54A	104.6 (3)
C13—C14—Ni1	72.00 (17)	C5—C4—C54A	130.6 (3)
C15—C14—H14	125.7	C6—C5—C4	129.2 (3)
C13—C14—H14	125.7	C6—C5—H5	115.4
Ni1—C14—H14	121.0	C4—C5—H5	115.4
C11—C15—C14	106.9 (3)	C5—C6—C8	124.3 (3)
C11—C15—Ni1	68.65 (18)	C5—C6—C7	120.9 (3)
C14—C15—Ni1	68.71 (17)	C8—C6—C7	114.7 (3)
C11—C15—H15	126.6	C52A—C51A—C3	103.7 (8)
C14—C15—H15	126.6	C52A—C51A—C55A	99.1 (10)
Ni1—C15—H15	127.6	C3—C51A—C55A	100.3 (9)
C22—C21—C26	118.1 (2)	C52A—C51A—H51A	117.0
C22—C21—P1	120.3 (2)	C53A—C52A—C51A	107.5 (9)
C26—C21—P1	121.5 (2)	C52A—C53A—C54A	106.7 (7)
C23—C22—C21	120.3 (3)	C54A—C55A—C51A	92.3 (8)
C23—C22—H22	119.8	C53A—C54A—C55A	99.9 (8)
C21—C22—H22	119.8	C53A—C54A—C4	101.6 (5)
C24—C23—C22	120.8 (3)	C55A—C54A—C4	102.2 (7)
C24—C23—H23	119.6	C52B—C51B—C3	99.6 (17)
C22—C23—H23	119.6	C52B—C51B—C55B	98.2 (18)
C25—C24—C23	119.8 (3)	C3—C51B—C55B	105.0 (11)
C25—C24—H24	120.1	C53B—C52B—C51B	107.1 (19)
C23—C24—H24	120.1	C52B—C53B—C54B	106.5 (15)
C24—C25—C26	119.7 (3)	C54B—C55B—C51B	92.3 (12)
C24—C25—H25	120.1	C53B—C54B—C55B	99.2 (11)
C26—C25—H25	120.1	C53B—C54B—C4	92.1 (8)
C25—C26—C21	121.2 (3)	C55B—C54B—C4	107.0 (9)
			
C1—Ni1—P1—C41	−52.45 (12)	C41—P1—C21—C26	−66.4 (2)
C11—Ni1—P1—C41	73.8 (4)	C31—P1—C21—C26	41.7 (2)
C14—Ni1—P1—C41	116.83 (14)	Ni1—P1—C21—C26	170.08 (19)
C12—Ni1—P1—C41	−177.3 (2)	C26—C21—C22—C23	1.3 (4)
C13—Ni1—P1—C41	157.32 (14)	P1—C21—C22—C23	179.0 (2)
C15—Ni1—P1—C41	81.63 (15)	C21—C22—C23—C24	0.9 (5)
C1—Ni1—P1—C21	68.98 (12)	C22—C23—C24—C25	−2.1 (5)
C11—Ni1—P1—C21	−164.7 (4)	C23—C24—C25—C26	1.2 (5)
C14—Ni1—P1—C21	−121.74 (14)	C24—C25—C26—C21	1.1 (5)
C12—Ni1—P1—C21	−55.9 (2)	C22—C21—C26—C25	−2.3 (4)
C13—Ni1—P1—C21	−81.26 (14)	P1—C21—C26—C25	−180.0 (2)
C15—Ni1—P1—C21	−156.95 (14)	C41—P1—C31—C36	2.6 (3)
C1—Ni1—P1—C31	−171.65 (13)	C21—P1—C31—C36	−108.7 (2)
C11—Ni1—P1—C31	−45.4 (4)	Ni1—P1—C31—C36	125.9 (2)
C14—Ni1—P1—C31	−2.38 (14)	C41—P1—C31—C32	−179.1 (2)
C12—Ni1—P1—C31	63.5 (2)	C21—P1—C31—C32	69.6 (2)
C13—Ni1—P1—C31	38.11 (15)	Ni1—P1—C31—C32	−55.8 (2)
C15—Ni1—P1—C31	−37.58 (15)	C36—C31—C32—C33	−0.4 (4)
C1—Ni1—C11—C15	135.0 (2)	P1—C31—C32—C33	−178.7 (2)
C14—Ni1—C11—C15	−36.8 (2)	C31—C32—C33—C34	1.0 (5)
C12—Ni1—C11—C15	−117.3 (3)	C32—C33—C34—C35	−0.5 (5)
C13—Ni1—C11—C15	−80.5 (2)	C33—C34—C35—C36	−0.7 (5)
P1—Ni1—C11—C15	10.1 (5)	C34—C35—C36—C31	1.3 (5)
C1—Ni1—C11—C12	−107.7 (2)	C32—C31—C36—C35	−0.8 (4)
C14—Ni1—C11—C12	80.5 (2)	P1—C31—C36—C35	177.5 (2)
C13—Ni1—C11—C12	36.8 (2)	C21—P1—C41—C42	7.7 (3)
C15—Ni1—C11—C12	117.3 (3)	C31—P1—C41—C42	−99.5 (3)
P1—Ni1—C11—C12	127.4 (4)	Ni1—P1—C41—C42	132.6 (2)
C15—C11—C12—C13	2.6 (4)	C21—P1—C41—C46	−173.8 (2)
Ni1—C11—C12—C13	−61.6 (2)	C31—P1—C41—C46	79.0 (3)
C15—C11—C12—Ni1	64.2 (2)	Ni1—P1—C41—C46	−48.9 (3)
C1—Ni1—C12—C13	−159.1 (2)	C46—C41—C42—C43	0.8 (5)
C11—Ni1—C12—C13	117.7 (3)	P1—C41—C42—C43	179.3 (2)
C14—Ni1—C12—C13	37.8 (2)	C41—C42—C43—C44	0.0 (5)
C15—Ni1—C12—C13	80.3 (2)	C42—C43—C44—C45	−1.0 (6)
P1—Ni1—C12—C13	−39.6 (3)	C43—C44—C45—C46	1.2 (6)
C1—Ni1—C12—C11	83.2 (2)	C44—C45—C46—C41	−0.4 (6)
C14—Ni1—C12—C11	−79.9 (2)	C42—C41—C46—C45	−0.6 (5)
C13—Ni1—C12—C11	−117.7 (3)	C2—C3—C4—C5	−3.8 (6)
C15—Ni1—C12—C11	−37.4 (2)	C51A—C3—C4—C5	−176.5 (6)
P1—Ni1—C12—C11	−157.29 (19)	C51B—C3—C4—C5	175.5 (9)
C11—C12—C13—C14	0.8 (4)	C2—C3—C4—C54B	−166.0 (6)
Ni1—C12—C13—C14	−59.1 (2)	C51A—C3—C4—C54B	21.2 (8)
C11—C12—C13—Ni1	59.9 (2)	C51B—C3—C4—C54B	13.3 (10)
C1—Ni1—C13—C12	34.2 (3)	C2—C3—C4—C54A	168.9 (4)
C11—Ni1—C13—C12	−38.6 (2)	C51A—C3—C4—C54A	−3.8 (7)
C14—Ni1—C13—C12	−118.9 (3)	C51B—C3—C4—C54A	−11.8 (10)
C15—Ni1—C13—C12	−81.0 (2)	C3—C4—C5—C6	177.3 (4)
P1—Ni1—C13—C12	158.0 (2)	C54B—C4—C5—C6	−24.0 (8)
C1—Ni1—C13—C14	153.1 (2)	C54A—C4—C5—C6	6.6 (8)
C11—Ni1—C13—C14	80.3 (2)	C4—C5—C6—C8	0.0 (6)
C12—Ni1—C13—C14	118.9 (3)	C4—C5—C6—C7	−177.4 (4)
C15—Ni1—C13—C14	37.9 (2)	C4—C3—C51A—C52A	−64.5 (9)
P1—Ni1—C13—C14	−83.1 (2)	C2—C3—C51A—C52A	122.1 (7)
C12—C13—C14—C15	−3.9 (3)	C4—C3—C51A—C55A	37.6 (8)
Ni1—C13—C14—C15	−64.4 (2)	C2—C3—C51A—C55A	−135.8 (7)
C12—C13—C14—Ni1	60.5 (2)	C3—C51A—C52A—C53A	67.7 (11)
C1—Ni1—C14—C15	7.4 (6)	C55A—C51A—C52A—C53A	−35.4 (11)
C11—Ni1—C14—C15	36.7 (2)	C51A—C52A—C53A—C54A	0.5 (11)
C12—Ni1—C14—C15	80.5 (2)	C52A—C51A—C55A—C54A	52.8 (10)
C13—Ni1—C14—C15	116.7 (3)	C3—C51A—C55A—C54A	−53.0 (10)
P1—Ni1—C14—C15	−132.0 (2)	C52A—C53A—C54A—C55A	35.2 (10)
C1—Ni1—C14—C13	−109.2 (5)	C52A—C53A—C54A—C4	−69.6 (9)
C11—Ni1—C14—C13	−80.0 (2)	C51A—C55A—C54A—C53A	−52.8 (9)
C12—Ni1—C14—C13	−36.2 (2)	C51A—C55A—C54A—C4	51.4 (10)
C15—Ni1—C14—C13	−116.7 (3)	C3—C4—C54A—C53A	71.0 (6)
P1—Ni1—C14—C13	111.32 (19)	C5—C4—C54A—C53A	−116.9 (6)
C12—C11—C15—C14	−5.0 (3)	C3—C4—C54A—C55A	−31.8 (8)
Ni1—C11—C15—C14	58.3 (2)	C5—C4—C54A—C55A	140.2 (8)
C12—C11—C15—Ni1	−63.3 (2)	C4—C3—C51B—C52B	59.8 (12)
C13—C14—C15—C11	5.5 (3)	C2—C3—C51B—C52B	−120.9 (13)
Ni1—C14—C15—C11	−58.2 (2)	C4—C3—C51B—C55B	−41.5 (15)
C13—C14—C15—Ni1	63.7 (2)	C2—C3—C51B—C55B	137.8 (10)
C1—Ni1—C15—C11	−58.3 (3)	C3—C51B—C52B—C53B	−72 (2)
C14—Ni1—C15—C11	119.1 (3)	C55B—C51B—C52B—C53B	35 (2)
C12—Ni1—C15—C11	38.6 (2)	C51B—C52B—C53B—C54B	1(3)
C13—Ni1—C15—C11	80.3 (2)	C52B—C51B—C55B—C54B	−53.8 (15)
P1—Ni1—C15—C11	−176.74 (18)	C3—C51B—C55B—C54B	48.5 (15)
C1—Ni1—C15—C14	−177.4 (2)	C52B—C53B—C54B—C55B	−36.9 (19)
C11—Ni1—C15—C14	−119.1 (3)	C52B—C53B—C54B—C4	70.7 (18)
C12—Ni1—C15—C14	−80.5 (2)	C51B—C55B—C54B—C53B	55.0 (12)
C13—Ni1—C15—C14	−38.8 (2)	C51B—C55B—C54B—C4	−40.0 (12)
P1—Ni1—C15—C14	64.1 (2)	C3—C4—C54B—C53B	−81.7 (8)
C41—P1—C21—C22	116.0 (2)	C5—C4—C54B—C53B	116.5 (7)
C31—P1—C21—C22	−135.9 (2)	C3—C4—C54B—C55B	18.6 (11)
Ni1—P1—C21—C22	−7.6 (2)	C5—C4—C54B—C55B	−143.2 (9)

### Hydrogen-bond geometry (Å, °)

**Table:**

*D*—H···*A*	*D*—H	H···*A*	*D*···*A*	*D*—H···*A*
C33—H33···Cg1^i^	0.93	2.98	3.648 (4)	130

Symmetry codes: (i) −*x*, −*y*+2, −*z*.
